# The impact of deployment experience and prior healthcare utilization on enrollment in a large military cohort study

**DOI:** 10.1186/1471-2288-13-90

**Published:** 2013-07-11

**Authors:** Jaime L Horton, Isabel G Jacobson, Alyson J Littman, John E Alcaraz, Besa Smith, Nancy F Crum-Cianflone

**Affiliations:** 1 Deployment Health Research Department, Naval Health Research Center, 140 Sylvester Rd, San Diego, California 92106, USA; 2 Department of Veterans Affairs Puget Sound Healthcare System, Seattle Epidemiologic Research and Information Center, 1660 South Columbian Way, Seattle, Washington, 98108, USA; 3 Department of Epidemiology, University of Washington, Seattle, Washington, USA; 4 San Diego State University, 5500 Campanile Dr, San Diego, California, 92182, USA

## Abstract

**Background:**

Longitudinal cohort studies are highly valued in epidemiologic research for their ability to establish exposure-disease associations through known temporal sequences. A major challenge in cohort studies is recruiting individuals representative of the targeted sample population to ensure the generalizability of the study’s findings.

**Methods:**

We evaluated nearly 350,000 invited subjects (from 2004-2008) of the Millennium Cohort Study, a prospective cohort study of the health of US military personnel, for factors prior to invitation associated with study enrollment. Multivariable logistic regression was utilized, adjusting for demographic and other confounders, to determine the associations between both deployment experience and prior healthcare utilization with enrollment into the study.

**Results:**

Study enrollment was significantly greater among those who deployed prior to and/or during the enrollment cycles or had at least one outpatient visit in the 12 months prior to invitation. Mental disorders and hospitalization for more than two days within the past year were associated with reduced odds of enrollment.

**Conclusions:**

These findings suggest differential enrollment by deployment experience and health status, and may help guide recruitment efforts in future studies.

## Background

Longitudinal studies are highly valued in epidemiology as they are designed to evaluate the temporal sequence of the exposure and disease pathway, and provide valuable information to inform inferences in these relationships [1-4]. Initial recruitment of participants is often very challenging due to privacy concerns, survey-related concerns (e.g., burden of the survey or sensitive content of questions), or lack of perceived benefit of participation [5]. The decision to enroll in a longitudinal study may be influenced by many factors. Characteristics that have been consistently observed to be associated with a higher probability of participation include female sex, older age, higher education and socioeconomic status [6-11].

Cohort studies often lack information on key factors for all members of the sampling frame, making it difficult to compare characteristics of enrolled participants with the original invited sample. If response to an invitation follows a random process [12], suggesting non-informative response, there may be little impact on study findings aside from reduced power. However, if the likelihood of response is directly related to the exposure or outcome under study, the findings may lack generalizability to the larger population from which the sample was drawn [13-16].

Military cohorts are characterized by unique experiences including deployments [17-21]. US military personnel are highly mobile and often more difficult to track and contact for long-term studies [22,23]. Deployment experience in support of the recent operations in Iraq and Afghanistan has not yet been studied as a determinant for enrollment in a longitudinal study. Additionally, prior health conditions may affect the probability of enrollment into an epidemiologic study, but most studies do not have these data on non-enrolled invitees. One study examining healthcare use in relation to enrollment of the first panel of the Millennium Cohort Study found some differences between responders and non-responders, but most differences were small in magnitude and it is unlikely that differential response due to health status substantially affected subsequent enrollment in Panel 1 [24]. Some studies of nonresponse have shown that individuals who receive more healthcare are more likely to respond to longitudinal studies [6,10,25-27]. However, few studies have had the ability to examine a US population because there is no universal health coverage and medical records are often not available for all invited subjects.

An important methodological strength of studies involving US military personnel is the availability of extensive electronic demographic, service-related, and health records. The Millennium Cohort Study is a prospective panel study designed to follow the long-term health and experiences of US military personnel for 21+ years during and after service, and has enrolled over 150,000 participants in the first three panels of the cohort from all service branches and components of the military [28,29]. The authors aimed to investigate whether deployment experiences and healthcare use were related to study enrollment, which may provide important methodologic information for future longitudinal studies.

## Methods

### Study population

The study population consisted of the entire study samples for Panels 2 and 3 of the Millennium Cohort Study, except those who refused to participate. Panel 1 was enrolled during 2001-2003 and consisted of 77,047 (30%) participants from a probability-based sample of 256,400 military personnel who had at least 1 year of service as of October 1, 2000; this panel has previously been studied in terms of nonresponse to enrollment [24] and follow-up [9] in relation to health status. For Panel 2, 150,000 randomly selected military personnel with 1-2 years of service as of October 2003 were invited. Women and Marines were oversampled in order to ensure adequate power to detect small differences in these subgroups. Postal and electronic mailings were sent inviting subjects to complete a questionnaire by paper or online from 1 June 2004 - 14 February 2006. The sample for Panel 3 included 200,000 randomly selected service members with 1-3 years of service as of October 2006, with oversampling for women and Marines; enrollment occurred from 5 June 2007 – 31 December 2008. Detailed descriptions of the methodology of the Millennium Cohort Study are available elsewhere [28,29].

Many efforts were made to maximize participation. Following a modified Dillman approach [30-32], introductory postcards, reminder emails, reminder postcards, and repeated survey mailings for non-responders were used to encourage enrollment. In addition to encouraging invitees to complete a survey, the postcards and other mailings were used to help verify the accuracy of contact information by using the US Postal Service’s “Return Service Requested” to obtain new forwarding addresses on undeliverable postal mail. Upon completion of a questionnaire on the web, participants were offered a free incentive of their choice (a hat, a shirt, or $5 phone card for Panel 2; a hat, a coin, or a $5 phone card for Panel 3) to thank them for their participation in the study.

### Outcome measure

The primary outcome was study enrollment. This measure was dichotomized as those who successfully completed a baseline questionnaire (enrollees) versus those approached regarding study enrollment who did not respond (non-responders). Persons who refused participation were excluded from these analyses. The use of electronic data records among all enrollees and non-responders included in this study was approved by the Institutional Review Board under Protocol Number NHRC.2000.0007.

### Main exposures

#### Deployment experience

Military-specific information was obtained from electronic personnel files provided by the Defense Manpower Data Center. In and out of theater dates were used to determine deployment experience in support of the recent operations in Iraq and Afghanistan. Individuals were categorized as those who did not deploy prior to the end of the enrollment cycle, those who deployed prior to the enrollment cycle only, those deployed during enrollment only, and those who deployed prior to and during the enrollment period.

#### Prior healthcare utilization

Medical data were obtained from the Military Health System Data Repository (MDR**)**. The MDR provided records from all outpatient encounters and inpatient encounters from Department of Defense military treatment facilities (MTFs) as well as TRICARE-approved civilian facilities. All primary diagnoses were recorded with *International Classification of Disease Manual, 9th Revision* (ICD-9) diagnostic codes. Healthcare data were restricted to active-duty personnel only because Reserve and National Guard personnel may not have consistent access to military-sponsored care, or may seek care outside of the military health system.

Prior healthcare utilization was determined by inpatient and outpatient records available from the MDR examining all documented use in the 12 months prior to study invitation for both panels (1 June 2003-1 June 2004 for Panel 2; 5 June 2006 – 5 June 2007 for Panel 3). Number of outpatient visits and cumulative days spent hospitalized were calculated for each person. Any principal diagnosis for mental disorders (yes, no; ICD-9 index codes 290-319) and any reported injuries (yes, no; ICD-9 index codes 800-959, 990-999) were determined. Reasons for outpatient care were also examined in a subanalysis using ICD-9 codes.

### Covariates

Covariates included sex, age, education, race/ethnicity, marital status, military pay grade, service branch, service component, and military occupation. These data were obtained from the Defense Manpower Data Center and examined at the time the sample was selected for invitation in the Millennium Cohort Study (October 2003 for Panel 2; October 2006 for Panel 3).

### Statistical analyses

Univariate analyses were conducted to evaluate the unadjusted associations between enrollment, the main exposures, and all covariates. Panels 2 and 3 were analyzed separately due to differences in study population and sampling scheme. Multivariable logistic regression models were built to calculate odds ratios of enrollment in relation to deployment experience, while adjusting for potential confounding by covariates. Additionally, a subanalysis was performed to examine participants who enrolled early (within the first month) compared to all other enrollees, given a prior study of Panel 1 subjects which found differences in these subgroups [33]. Separate logistic regression models were built to calculate the odds of enrollment in relation to prior healthcare utilization among active-duty service members only (see Prior healthcare utilization), adjusting for all demographic and service-related covariates described. Another subanalysis investigated ICD-9 codes to determine types of outpatient care used in the year prior to invitation and the most common reasons for presenting for care. Regression diagnostics were conducted to identify multicollinearity using a variance inflation factor of 4 or greater [34]. Data management and statistical analyses were performed using SAS software, version 9.3 (SAS Institute, Inc., Cary, North Carolina).

## Results

Of the 150,000 service members invited to Panel 2, 31,110 (21%) enrolled in the study; and of the 200,000 service members invited to Panel 3, 43,440 (22%) enrolled in the study. There were 497 Panel 2 invitees and 329 Panel 3 invitees who explicitly refused to participate in the Cohort and were thus excluded from analyses, leaving study populations of 149,503 and 199,671 invitees to Panels 2 and 3, respectively. The invited samples consisted of primarily male, younger active-duty members who were never married and had some college education or less. Approximately 50% and 58% of Panels 2 and 3 enrollees, respectively, deployed in support of the recent operations. In both panels, active-duty personnel with four or more outpatient visits were proportionally more likely to enroll in the study. The majority of active-duty service members who enrolled in the study did not have any overnight hospitalizations in the year prior to invitation. The proportion of individuals with mental disorder diagnoses were very similar between enrollees and the invited sample, while the proportion of individuals with injuries was slightly higher among enrollees compared with the invited sample (Table 1).

**Table 1:** Characteristics of invited subjects, enrollees, and non-responders in the Millennium Cohort Study by panel (2004-2008)

	**Panel 2 (2004-2006)**^**a**^			**Panel 3 (2007-2008)**^**b**^		
	**Invited**^**d**^	**Enrollees**^**e**^	**Non-Responders**^**f**^	**Invited**^**d**^	**Enrollees**^**e**^	**Non-Responders**^**f**^
	**(N=149,503)**	**(N=31,110)**	**(N=118,393)**	**(N=199,671)**	**(N=43,440)**	**(N=156,231)**
**Characteristic**^**c**^	**n (%)**	**n (%)**	**n (%)**	**n (%)**	**n (%)**	**n (%)**
Deployment experience^g^*						
None	76,087 (50.9)	15,682 (50.4)	60,405 (51.0)	75,014 (37.6)	18,168 (41.8)	56,846 (36.4)
Prior to enrollment only	18,757 (12.6)	3,994 (12.8)	14,763 (12.5)	46,037 (23.1)	9,268 (21.3)	36,769 (23.5)
During enrollment only	37,236 (24.9)	8,282 (26.6)	28,954 (24.5)	45,921 (23.0)	9,580 (22.1)	36,341 (23.3)
Prior to and during enrollment	17,423 (11.7)	3,152 (10.1)	14,271 (12.0)	32,699 (16.4)	6,424 (14.8)	26,275 (16.8)
Number of outpatient visits^h^*						
None	11,396 (12.6)	1,421 (7.6)	9,975 (13.8)	18,426 (12.1)	2,494 (7.2)	15,932 (13.6)
1-3 visits	24,047 (26.5)	4,100 (22.0)	19,947 (27.7)	39,226 (25.8)	7,641 (22.2)	31,585 (26.9)
4-12 visits	33,099 (36.5)	7,163 (38.4)	25,936 (36.0)	56,426 (37.2)	13,723 (39.8)	42,703 (36.4)
>12 visits	22,164 (24.4)	5,962 (32.0)	16,202 (22.5)	37,724 (24.9)	10,590 (30.7)	27,134 (23.1)
Cumulative days hospitalized^h^*						
None	85,083 (93.8)	17,335 (93.0)	67,748 (94.0)	143,135 (94.3)	32,253 (93.6)	110,882 (94.5)
1-2 days	2,770 (3.1)	696 (3.7)	2,074 (2.9)	4,242 (2.8)	1,148 (3.3)	3,094 (2.6)
> 2 days	2,853 (3.1)	615 (3.3)	2,238 (3.1)	4,425 (2.9)	1,047 (3.0)	3,378 (2.9)
Any diagnosed mental disorder^i^						
No	79,984 (88.2)	16,377 (87.8)	63,607 (88.3)	133,992 (88.3)	30,443 (88.4)	103,549 (88.2)
Yes	10,722 (11.8)	2,269 (12.2)	8,453 (11.7)	17,810 (11.7)	4,005 (11.6)	13,805 (11.8)
Any reported injury^j^*						
No	64,912 (71.6)	12,911 (69.2)	52,001 (72.2)	112,173 (73.9)	24,768 (71.9)	87,405 (74.5)
Yes	25,794 (28.4)	5,735 (30.8)	20,059 (27.8)	39,629 (26.1)	9,680 (28.1)	29,949 (25.5)
Sex*						
Male	111,781 (74.8)	19,167 (61.6)	92,614 (78.2)	152,029 (76.1)	27,941 (64.3)	124,088 (79.4)
Female	37,722 (25.2)	11,943 (38.4)	25,779 (21.8)	47,642 (23.9)	15,499 (35.7)	32,143 (20.6)
Age (years)*						
17-20	49,372 (33.0)	9,317 (30.0)	40,055 (33.8)	35,767 (17.9)	6,005 (13.8)	29,762 (19.0)
21-22	35,666 (23.9)	6,693 (21.5)	28,973 (24.5)	74,159 (37.1)	13,916 (32.0)	60,243 (38.6)
23-24	24,936 (16.7)	5,248 (16.9)	19,688 (16.6)	41,549 (20.8)	9,108 (21.0)	32,441 (20.8)
>24	39,529 (26.4)	9,852 (31.7)	29,677 (25.1)	48,196 (24.1)	14,411 (33.2)	33,785 (21.6)
Education*						
Some college or less	131,935 (88.2)	25,752 (82.8)	106,183 (89.7)	179,919 (90.1)	36,287 (83.5)	143,632 (91.9)
Bachelors +	11,347 (7.6)	4,291 (13.8)	7,056 (6.0)	14,344 (7.2)	5,856 (13.5)	8,488 (5.4)
Unknown	6,221 (4.2)	1,067 (3.4)	5,154 (4.4)	5,408 (2.7)	1,297 (3.0)	4,111 (2.6)
Marital status*						
Never married	110,876 (74.2)	21,597 (69.4)	89,279 (75.4)	136,727 (68.5)	26,839 (61.8)	109,888 (70.3)
Married	35,688 (23.9)	8,708 (28.0)	26,980 (22.8)	59,564 (29.8)	15,574 (35.9)	43,990 (28.2)
Divorced, separated, or widowed	2,604 (1.7)	711 (2.3)	1,893 (1.6)	3,380 (1.7)	1,027 (2.4)	2,353 (1.5)
Unknown	335 (0.2)	94 (0.3)	241 (0.2)	0 (0.0)	0 (0.0)	0 (0.0)
Race/ethnicity*						
White, non- Hispanic	99,362 (66.5)	22,176 (71.3)	77,186 (65.2)	138,261 (69.2)	31,363 (72.2)	106,898 (68.4)
Black, non- Hispanic	23,060 (15.4)	3,612 (11.6)	19,448 (16.4)	26,888 (13.5)	4,900 (11.3)	21,988 (14.1)
Asian/Pacific Islander	5,857 (3.9)	1,537 (4.9)	4,320 (3.6)	9,821 (4.9)	2,451 (5.6)	7,370 (4.7)
Hispanic	16,995 (11.4)	3,142 (10.1)	13,853 (11.7)	18,926 (9.5)	3,407 (7.8)	15,519 (9.9)
Other	2,260 (1.5)	601 (1.9)	1,659 (1.4)	5,775 (2.9)	1,319 (3.0)	4,456 (2.9)
Unknown	1,969 (1.3)	42 (0.1)	1,927 (1.6)	0 (0.0)	0 (0.0)	0 (0.0)
Military pay grade*						
Enlisted	139,848 (93.5)	27,482 (88.3)	112,366 (94.9)	187,964 (94.1)	38,455 (88.5)	149,509 (95.7)
Officer	9,655 (6.5)	3,628 (11.7)	6,027 (5.1)	11,707 (5.9)	4,985 (11.5)	6,722 (4.3)
Service component*						
Reserve/Guard	58,711 (39.3)	12,459 (40.0)	46,252 (39.1)	47,869 (24.0)	8,992 (20.7)	38,877 (24.9)
Active-Duty	90,792 (60.7)	18,651 (60.0)	72,141 (60.9)	151,802 (76.0)	34,448 (79.3)	117,354 (75.1)
Branch of service*						
Army	61,442 (41.1)	14,995 (48.2)	46,447 (39.2)	81,067 (40.6)	15,798 (36.4)	65,269 (41.8)
Navy/Coast Guard	28,110 (18.8)	5,263 (16.9)	22,847 (19.3)	35,893 (18.0)	7,922 (18.2)	27,971 (17.9)
Marine Corps	29,939 (20.0)	2,576 (8.3)	27,363 (23.1)	49,942 (25.0)	6,802 (15.7)	43,140 (27.6)
Air Force	30,012 (20.1)	8,276 (26.6)	21,736 (18.4)	32,769 (16.4)	12,918 (29.7)	19,851 (12.7)
Military occupation*						
Combat specialist	47,502 (31.8)	6,833 (22.0)	40,669 (34.4)	44,437 (22.3)	6,333 (14.6)	38,104 (24.4)
Electronic equipment repairer	9,333 (6.2)	2,490 (8.0)	6,843 (5.8)	13,935 (7.0)	3,867 (8.9)	10,068 (6.4)
Comm/intel	10,529 (7.0)	2,678 (8.6)	7,851 (6.6)	16,737 (8.4)	4,079 (9.4)	12,658 (8.1)
Healthcare	9,809 (6.6)	3,444 (11.1)	6,365 (5.4)	14,144 (7.1)	4,477 (10.3)	9,667 (6.2)
Other technical and allied	3,487 (2.3)	935 (3.0)	2,552 (2.2)	5,904 (3.0)	1,642 (3.8)	4,262 (2.7)
Functional support/admin	17,841 (11.9)	4,721 (15.2)	13,120 (11.1)	28,018 (14.0)	7,589 (17.5)	20,429 (13.1)
Electrical/mechanical equipment repairer	22,350 (14.9)	4,171 (13.4)	18,179 (15.4)	34,990 (17.5)	7,087 (16.3)	27,903 (17.9)
Craft worker	5,398 (3.6)	1,034 (3.3)	4,364 (3.7)	7,032 (3.5)	1,330 (3.1)	5,702 (3.7)
Service and supply	16,210 (10.8)	3,219 (10.4)	12,991 (11.0)	24,281 (12.2)	4,144 (9.5)	20,137 (12.9)
Students, trainees, other	7,044 (4.7)	1,585 (5.1)	5,459 (4.6)	5,829 (2.9)	1,689 (3.9)	4,140 (2.7)
Unknown	0 (0.0)	0 (0.0)	0 (0.0)	4,364 (2.2)	1,203 (2.8)	3,161 (2.0)

*Chi-square test of enrollees vs. non-responders was significant at the *P* < 0.05 level for both panels.

^a^The Panel 2 enrollment cycle was from 1 June 2004 to 14 February 2006.

^b^The Panel 3 enrollment cycle was from 5 June 2007 to 31 December 2008.

^c^Characteristics were evaluated at the time each sample was drawn (October 2003 for Panel 2; October 2006 for Panel 3).

^d^Invited subjects are all those approached regarding study enrollment who did not refuse to participate in the Millennium Cohort Study.

^e^Enrolled subjects are all eligible, invited subjects who successfully completed a baseline questionnaire during the enrollment cycle.

^f^Non-responders include all those who were approached but did not respond or complete a baseline questionnaire during the enrollment cycle.

^g^Any deployment experience in support of the operations in Iraq and Afghanistan.

^h^Any cause healthcare utilization in the 12 months prior to the first day of the enrollment cycle, among active-duty personnel only.

^i^Any reported principal diagnosis for a mental disorder, as defined by ICD-9 codes 290-319, among active-duty personnel only.

^j^Any reported principal diagnosis for an injury, as defined by ICD-9 codes 800-959, 990-999, among active-duty personnel only.

After excluding 17,434 (8,137 Panel 2; 9,292 Panel 3) invitees for missing or unknown demographic information, there were 141,366 and 190,379 individuals in Panels 2 and 3, respectively, included in adjusted analyses of enrollment status in relation to deployment experience (Table 2). Among those excluded due to missing demographic information, 20% (14% Panel 2; 25% Panel 3) enrolled in the study (data not shown). Education was the most common missing demographic variable in both panels, followed by marital status in Panel 2 and duty occupation in Panel 3. The greater proportion of non-responders missing demographic information may have been due to recent separation from service, since those no longer serving may have missing values in the database. Previous investigations of nonresponse among Panel 1 members showed separation from service as a predictor of nonresponse [9]. Exclusion of a larger proportion of non-responders vs. responders may have biased our estimates slightly, but this is thought to be nonappreciable given the large sample sizes of both panels.

Deployment experience prior to and/or during enrollment was associated with increased odds (8-18%) of enrolling in Panel 2 relative to nondeployers. Individuals with deployment experience during enrollment as well as prior to *and* during enrollment were 5% more likely to enroll in Panel 3 than those with no deployment experience. Females, older age groups, married service members, those with higher education, on active-duty status, and officers had significantly higher odds of enrollment in both panels. Conversely, non-Hispanic blacks and Hispanics had reduced odds of enrollment when compared to non-Hispanic whites. Navy or Coast Guard members had reduced odds of enrolling in Panel 2, but increased odds of enrolling in Panel 3 when compared with Army personnel. Marine Corps members had significantly lower odds of enrolling in both panels. Odds of enrollment among Air Force members were no different than the Army for Panel 2, while the odds of Air Force members enrolling in Panel 3 were more than twice as high as the Army. Service members in the combat specialist occupation were the least likely of all occupational categories to enroll in the study.

**Table 2:** **Adjusted**^**a **^**odds of enrollment status in the Millennium Cohort Study by panel (2004-2008)**

	**Panel 2 (2004-2006)**		**Panel 3 (2007-2008)**	
	**N = 141,366**^**b**^		**N = 190,379**^**c**^	
**Characteristic**^**d**^	**OR**	**(95% CI)**	**OR**	**95% CI**
Deployment experience^e^				
None	1.00	Ref	1.00	Ref
Prior to enrollment only	1.11*	1.06, 1.16	1.02	0.99, 1.06
During enrollment only	1.18*	1.14, 1.22	1.05*	1.02, 1.08
Prior to and during enrollment	1.08*	1.03, 1.13	1.05*	1.02, 1.09
Sex				
Male	1.00	Ref	1.00	Ref
Female	1.91*	1.85, 1.97	1.77*	1.73, 1.82
Age (years)				
17-20	1.00	Ref	1.00	Ref
21-22	0.98	0.95, 1.02	1.04*	1.01, 1.08
23-24	1.19*	1.14, 1.24	1.10*	1.05, 1.14
>24	1.36*	1.31, 1.42	1.44*	1.38, 1.50
Education				
Some college or less	1.00	Ref	1.00	Ref
Bachelors or higher	1.49*	1.39, 1.60	1.47*	1.38, 1.58
Marital status				
Never married	1.00	Ref	1.00	Ref
Married	1.16*	1.12, 1.20	1.21*	1.18, 1.24
Divorced, separated, or widowed	1.06	0.96, 1.17	1.10*	1.02, 1.20
Race/ethnicity				
White, non-Hispanic	1.00	Ref	1.00	Ref
Black, non-Hispanic	0.52*	0.50, 0.54	0.64*	0.62, 0.66
Asian/Pacific Islander	0.98	0.92, 1.04	0.99	0.94, 1.05
Hispanic	0.79*	0.76, 0.82	0.81*	0.77, 0.84
Other	1.20*	1.08, 1.33	0.95	0.88, 1.01
Military pay grade				
Enlisted	1.00	Ref	1.00	Ref
Officer	1.48*	1.36, 1.61	1.35*	1.25, 1.45
Service component				
Reserve/Guard	1.00	Ref	1.00	Ref
Active-Duty	1.29*	1.25, 1.33	1.27*	1.24, 1.31
Branch of service				
Army	1.00	Ref	1.00	Ref
Navy/Coast Guard	0.74*	0.71, 0.77	1.06*	1.02, 1.10
Marine Corps	0.38*	0.36, 0.39	0.80*	0.77, 0.83
Air Force	1.01	0.98, 1.05	2.30*	2.23, 2.37
Military occupation				
Combat specialist	1.00	Ref	1.00	Ref
Electronic equipment repairer	1.73*	1.64, 1.83	1.80*	1.72, 1.89
Communications/intelligence	1.57*	1.49, 1.66	1.58*	1.51, 1.66
Healthcare	1.90*	1.80, 2.00	1.72*	1.63, 1.80
Other technical and allied	1.67*	1.54, 1.82	1.87*	1.75, 1.99
Functional support/administration	1.71*	1.63, 1.79	1.83*	1.75, 2.00
Electrical/mechanical equipment repairer	1.28*	1.22, 1.34	1.44*	1.39, 1.50
Craft worker	1.32*	1.22, 1.43	1.36*	1.27, 1.45
Service and supply	1.25*	1.19, 1.31	1.25*	1.19, 1.31
Students, trainees, other	1.36*	1.27, 1.46	1.69*	1.58, 1.80

OR, odds ratio; CI, confidence interval; Ref, reference category.

*Indicates significance at the *P* < 0.05 level.

^a^Models were adjusted for all variables included in the table.

^b^There were 8,137 service members excluded due to missing or unknown demographic information.

^c^There were 9,292 service members excluded due to missing or unknown demographic information.

^d^Characteristics were evaluated at the time each sample was drawn (October 2003 for Panel 2; October 2006 for Panel 3).

^e^Any deployment experience in support of the operations in Iraq and Afghanistan.

Results of the subanalysis (data not shown) revealed that later enrollees shared many characteristics with the non-responders in this population, while the early enrollees tended to be female, older, and more educated. Interestingly, enrollees with any deployment experience were significantly more likely to enroll later in the survey cycle than those with no deployment experience.

Analyses of prior healthcare utilization were restricted to active-duty personnel, leaving study populations of 86,401 from Panel 2 and 144,954 from Panel 3. In adjusted analyses, active-duty personnel who had at least one outpatient visit in the 12 months prior to study invitation had significantly higher odds of enrolling in Panel 2 and Panel 3 than those who did not have any reported outpatient visits (Table 3). The odds of enrolling in Panel 3 increased as the number of outpatient visits increased. Invited subjects with more than 12 outpatient visits were significantly more likely to enroll in Panel 3 than those with no visits (odds ratio [OR], 1.44; 95% confidence interval [CI], 1.36-1.52) (Table 3) and those with 1-3 visits (OR, 1.14; 95% CI, 1.10-1.19), but were no different than those with 4-12 visits (OR, 1.03; 95% CI, 0.99-1.06) (data not shown). The most common types of outpatient care sought by enrollees included routine medical examinations or screenings (17%), other consultations or follow-up exams (11%), musculoskeletal conditions (10%), or procedures and aftercare for a previously treated condition (9%) (data not shown).

**Table 3:** Adjusted odds of enrollment status with prior healthcare utilization in the Millennium Cohort Study by panel, among active-duty personnel

	**Panel 2 (2004-2006)**		**Panel 3 (2007-2008)**	
	**N = 86,401**^**a**^		**N = 144,954**^**a**^	
**Exposure**^**b,c**^	**OR**	**95% CI**	**OR**	**95% CI**
Number of outpatient visits^d^				
None	1.00	Ref	1.00	Ref
1-3 visits	1.13*	1.05, 1.20	1.26*	1.20, 1.33
4-12 visits	1.17*	1.10, 1.25	1.41*	1.34, 1.48
>12 visits	1.14*	1.06, 1.23	1.44*	1.36, 1.52
Cumulative days hospitalized^d^				
None	1.00	Ref	1.00	Ref
1-2 days	0.88*	0.80, 0.97	0.96	0.89, 1.03
More than 2 days	0.75*	0.68, 0.83	0.89*	0.83, 0.96
Any diagnosed mental disorder^e^				
No	1.00	Ref	1.00	Ref
Yes	0.79*	0.75, 0.83	0.89*	0.85, 0.93
Any reported injury^f^				
No	1.00	Ref	1.00	Ref
Yes	1.03	0.99, 1.07	1.10*	1.07, 1.13

OR, odds ratio; CI, confidence interval; Ref, reference category.

*Indicates significance at the *P* < 0.05 level.

^a^Only active-duty personnel with complete demographic and covariate information were included.

^b^Exposures were evaluated during the 12 months prior to each enrollment cycle (1 June 2003 – 1 June 2004 for Panel 2 and 5 June 2006 – 5 June 2007 for Panel 3).

^c^Each exposure was modeled separately with enrollment status and adjusted for sex, age, education, marital status, race/ethnicity, service branch, deployment experience, and military occupation. Military pay grade was excluded from these models due to small numbers of officers in these young, active-duty populations.

^d^Any cause healthcare utilization in the 12 months prior to the first day of the enrollment cycle.

^e^Any reported principal diagnosis for a mental disorder, as defined by ICD-9 codes 290-319.

^f^Any reported principal diagnosis for an injury, as defined by ICD-9 codes 800-959, 990-999.

Conversely, those who were hospitalized for more than two days had reduced odds of enrolling in Panel 2 (OR: 0.75, 95% CI: 0.68-0.83) and Panel 3 (OR: 0.89, 95% CI: 0.83-0.96) compared to those with no hospitalizations in the year prior to enrollment. Service members with prior reported mental disorder diagnoses also had significantly reduced odds of enrolling in the Millennium Cohort Study. Prior reported injuries were not significantly associated with enrollment in Panel 2, while Panel 3 invitees with prior injuries were significantly more likely to enroll (OR: 1.10; 95% CI: 1.07-1.13) than those with no documented healthcare use for injuries in the year prior to invitation.

## Discussion

This study provides insight into key determinants that were associated with enrollment of young military personnel in a long-term health study. The authors suspected that the deployed population would be less likely to enroll as they are often more mobile and potentially more difficult to contact, busier with training, unable to respond from their deployed location, or overwhelmed with other health- or deployment-related surveys, but in fact found the opposite. Additionally, active-duty service members who had at least one outpatient visit prior to invitation had significantly higher odds of enrolling in the study, whereas those with hospital stays longer than two days and prior mental disorders were less likely to enroll. Although it is reassuring that we are able to enroll service members with deployment experience and capture this unique population’s experiences, it is important to realize that persons with mental disorders and inpatient hospitalizations prior to enrollment were underrepresented in our sample.

In voluntary longitudinal studies, the most common reason for participation is the direct perceived benefit of the study for the individual [35]. US military personnel with deployment experience were significantly more likely to enroll, perhaps in hopes of having their voice heard and military experiences reported. On the other hand, combat specialists were less likely than all other occupational categories to enroll in the Millennium Cohort Study. It is possible that there is a difference in response among those who experience combat while deployed and those who do not, but we were unable to capture combat experiences in this study. Further study regarding nonresponse of this occupational group is warranted.

The finding that enrollees were more likely to have prior outpatient healthcare visits was further investigated by examining the reasons for seeking care. Enrollees who presented for outpatient care in the year prior to invitation mostly did so for routine and preventive care, and not for more severe conditions. Findings from the study of Panel 1 invitees showed that individuals who sought outpatient care for certain conditions were more likely to enroll in the Millennium Cohort Study; however the magnitude of the associations were small and whether these visits were for preventive care versus treatment seeking was not investigated in this prior study [24]. Those engaged in routine and preventive care may have more interest in participating in a study focused on health outcomes, or may be among the “worried well”. Conversely, invitees who had been hospitalized more than two days in the year prior to enrollment were significantly less likely to participate in the study. Those hospitalized may represent a population in poorer health who may be less reachable or less likely to prioritize the completion of a survey. This finding was not observed in Panel 1 [24], potentially because of differences in demographic characteristics, years of service, timing of enrollment, sampling strategy between panels, and the method in which the exposure was measured. Panels 2 and 3 are comprised of younger service members with 1-3 years of service invited to the cohort during a heightened tempo of the recent wars in Iraq and Afghanistan, and the odds of response were examined by cumulative days of hospitalization; while Panel 1 represents an older population who joined the service prior to September 11, 2001, with an oversampling for those who previously served in Southwest Asia, Bosnia, and Kosovo, and odds of response in this study were examined by hospitalization for any cause (yes vs. no). When the mean number of days hospitalized were examined among Panel 1, non-responders had significantly more mean days of hospitalization compared with responders (*p* = 0.034) [24], indicating a similar pattern to the present study where more time hospitalized was associated with nonresponse. Nonetheless, these findings have implications to other cohort studies interested in examining health outcomes or debilitating diseases.

Invited subjects in both panels with prior mental disorder diagnoses were significantly less likely to enroll in the study, similar to those in Panel 1 [24]. Mental disorders, particularly mood disorders, have been shown to be associated with more impairment in physical, social, and role functioning than common medical disorders [36,37]. Such factors may impact a subject’s ability or desire to respond to voluntary studies. The Millennium Cohort Study, like several others [27,38,39], may therefore underrepresent individuals with baseline mental disorders.

Seeking healthcare for an injury in the year before invitation was associated with a significant, albeit, small increase in the odds of enrollment in Panel 3. Injuries are associated with increased impairment in physical functioning, but there may be little impairment in other health components such as social or role functioning, perception of general health, or mental health in this young, active-duty population. It is possible that those with injuries felt more inclined to document their experiences by responding to the survey.

Consistent with other studies [6-9], the present study found that female sex, white race, more education, and older age are associated with better rates of response. In addition, those with a higher military pay grade (a surrogate for socioeconomic status) and those on active-duty status were more likely to enroll in this cohort. Marine Corps members had significantly reduced odds of participating in this study; a group that is often difficult to engage and therefore oversampled in the Millennium Cohort Study. These analyses are useful in guiding current and future recruitment efforts to reach these subgroups and encourage their participation through tailored messages.

When interpreting these findings, several important limitations should be understood. First, although all invitees were approached to enroll in the study via postal mail and email invitations, precise data on the number and types of contacts received were not available and may have varied between invitees. Further, we could not evaluate the characteristics of those who refused study participation in the current analysis. Second, analyses related to healthcare utilization were restricted to active-duty personnel since inactive Reserve/Guard members may not have equal access to care. Thus, we were unable to make inferences regarding the association between healthcare utilization and enrollment in this important component of the military. The use of ICD-9 codes for certain health outcomes such as mental disorders and injuries may not represent the true burden of disease since individuals experiencing symptoms do not always seek care. Some misclassification in medical data records or ICD-9 codes is also possible, although this was likely to have been nondifferential with respect to enrollment status. Also, behavioral and other health characteristics or potential confounders could not be included since this study was limited to electronic data captured by the military health system. Finally, while we were able to ascertain deployment status, we were not able to gather any information on experiences during deployment, such as combat experience or in-theater injuries, that may have also been associated with subsequent enrollment.

Despite these limitations, this study has important strengths. This study provided a unique opportunity to investigate characteristics of enrollees and non-responders to a cohort study, and allowed comparison of nearly the entire invited sample with those who enrolled. Data on demographics, education, occupation, healthcare use, and military deployments were available for the entire study sample prior to invitation. The Millennium Cohort consists of members from all branches and components of the Armed Forces, yielding a large sample size with adequate power to detect meaningful differences among subgroups of the population. In addition, military healthcare is equally accessible to all active-duty personnel, so all enrollees and non-responders theoretically had equal access to care, reducing the potential for bias due to socioeconomic factors in the associations between healthcare utilization and enrollment.

## Conclusions

In all cohort studies, a comprehensive assessment of nonresponse is necessary for proper interpretation of study findings as well as understanding internal and external validity. Important factors associated with nonresponse were identified in this study which may be applied to other cohort studies. Researchers should be aware that individuals requiring inpatient care or diagnosed with mental disorders may be less likely to enroll in voluntary studies, and various sampling and recruitment techniques should be considered. The present study also identified key determinants associated with a greater likelihood of enrollment in a military cohort, such as deployment experience, higher pay grade, and service component, that may guide future research strategies. Continuing to investigate differential response by demographic, service-related, and health-related characteristics and applying new recruitment techniques may provide important methodologic information for future longitudinal studies and aid in increasing response rates and reducing nonresponse bias.

## Competing interests

All authors declare that they have no competing interests.

## Authors’ contributions

JH had full access to all of the data in the study and takes responsibility for the integrity of the data and the accuracy of the data analysis. All authors were involved in the study concept and design. JH and IJ worked on the data analysis and all authors were involved in the analysis plans and interpretation of data and results. All authors contributed to the writing of the manuscript and critically reviewed for important intellectual content. All authors reviewed and approved the final version to be published.

## Pre-publication history

The pre-publication history for this paper can be accessed here:

http://www.biomedcentral.com/1471-2288/13/90/prepub

## Disclaimer

This represents report 12-54, supported by the Department of Defense, under work unit no. 60002. The views expressed in this article are those of the authors and do not reflect the official policy or position of the Department of the Navy, Department of the Army, Department of the Air Force, Department of Defense, Department of Veterans Affairs, or the US Government. This research has been conducted in compliance with all applicable federal regulations governing the protection of human subjects in research (Protocol NHRC.2000.0007).
